# The temperate *Burkholderia* phage AP3 of the *Peduovirinae* shows efficient antimicrobial activity against *B. cenocepacia* of the IIIA lineage

**DOI:** 10.1007/s00253-016-7924-7

**Published:** 2016-10-21

**Authors:** Bartosz Roszniowski, Agnieszka Latka, Barbara Maciejewska, Dieter Vandenheuvel, Tomasz Olszak, Yves Briers, Giles S. Holt, Miguel A. Valvano, Rob Lavigne, Darren L. Smith, Zuzanna Drulis-Kawa

**Affiliations:** 1 Institute of Genetics and Microbiology, University of Wroclaw, Przybyszewskiego 63/77, 51-148 Wroclaw, Poland; 2 Laboratory of Gene Technology, KU Leuven, Kasteelpark Arenberg 21, box 2462, 3001 Leuven, Belgium; 3 Department of Applied Biosciences, Ghent University, Valentin Vaerwyckweg 1, 9000 Ghent, Belgium; 4 Applied Sciences, University of Northumbria, Ellison Building EBD222, Newcastle upon Tyne, NE1 8ST UK; 5 Center for Experimental Medicine, Queen’s University of Belfast, 97 Lisburn Rd., Belfast, BT9 7BL UK

**Keywords:** Temperate phage, *Peduovirinae*, *Burkholderia cepacia* lineage IIIA

## Abstract

*Burkholderia* phage AP3 (vB_BceM_AP3) is a temperate virus of the *Myoviridae* and the *Peduovirinae* subfamily (*P2likevirus* genus). This phage specifically infects multidrug-resistant clinical *Burkholderia cenocepacia* lineage IIIA strains commonly isolated from cystic fibrosis patients. AP3 exhibits high pairwise nucleotide identity (61.7 %) to *Burkholderia* phage KS5, specific to the same *B. cenocepacia* host, and has 46.7–49.5 % identity to phages infecting other species of *Burkholderia*. The lysis cassette of these related phages has a similar organization (putative antiholin, putative holin, endolysin, and spanins) and shows 29–98 % homology between specific lysis genes, in contrast to *Enterobacteria* phage P2, the hallmark phage of this genus. The AP3 and KS5 lysis genes have conserved locations and high amino acid sequence similarity. The AP3 bacteriophage particles remain infective up to 5 h at pH 4–10 and are stable at 60 °C for 30 min, but are sensitive to chloroform, with no remaining infective particles after 24 h of treatment. AP3 lysogeny can occur by stable genomic integration and by pseudo-lysogeny. The lysogenic bacterial mutants did not exhibit any significant changes in virulence compared to wild-type host strain when tested in the *Galleria mellonella* moth wax model. Moreover, AP3 treatment of larvae infected with *B. cenocepacia* revealed a significant increase (*P* < 0.0001) in larvae survival in comparison to AP3-untreated infected larvae. AP3 showed robust lytic activity, as evidenced by its broad host range, the absence of increased virulence in lysogenic isolates, the lack of bacterial gene disruption conditioned by bacterial tRNA downstream integration site, and the absence of detected toxin sequences. These data suggest that the AP3 phage is a promising potent agent against bacteria belonging to the most common *B. cenocepacia* IIIA lineage strains.

**Electronic supplementary material:**

The online version of this article (doi:10.1007/s00253-016-7924-7) contains supplementary material, which is available to authorized users.

## Introduction

Gram-negative, non-fermentative bacilli of the *Burkholderia cepacia* complex (BCC) are inherently difficult to eradicate clinically. They are dangerous opportunistic pathogens that infect patients suffering from cystic fibrosis (CF). The BCC includes 17 closely related bacterial species (*B. cepacia*, *Burkholderia multivorans*, *Burkholderia cenocepacia*, *Burkholderia stabilis*, *Burkholderia vietnamiensis*, *Burkholderia dolosa*, *Burkholderia ambifaria*, *Burkholderia anthina*, *Burkholderia pyrrocinia*, *Burkholderia ubonensis*, *Burkholderia latens*, *Burkholderia diffusa*, *Burkholderia arboris*, *Burkholderia seminalis*, *Burkholderia metallica*, *Burkholderia contaminans*, and *Burkholderia lata*), which can only be discriminated from each other using molecular methods (Drevinek and Mahenthiralingam 2010; Medina-Pascual et al. 2012). The BCC bacteria survive for long periods in moist environments and can cause hospital outbreaks especially in immunocompromised patients; they also contaminate pharmaceutical products and water supplies (Gilligan et al. 2003). The most prevalent isolates in CF patients, *B. multivorans* and *B. cenocepacia*, are responsible for 85–97 % of infections (Drevinek and Mahenthiralingam 2010). *B. cenocepacia* also infects patients suffering from chronic granulomatous disease (CGD; Bylund et al. 2005). Lung infection of CF patients by BCC leads to “cepacia syndrome,” an acute, necrotizing pneumonia leading to death (Isles et al. 1984). Based on *recA* gene polymorphisms, *B. cenocepacia* isolates can be subdivided into four lineages. Lineages IIIA–D and IIIA/IIID are exclusively found in clinical samples, IIIC occurs only in soil, and IIIB occurs in both environmental and clinical samples (Mahenthiralingam et al. 2000; Vandamme et al. 2003; Manno et al. 2004). *B. cenocepacia* has multiple virulence factors, including cepacian exopolysaccharide (crucial for chronic infections), adhesins, pili and flagella, biofilm, and type III and IV secretion systems, recently reviewed in detail (Leitão et al. 2010; Casey and McClean 2015). The *B. cenocepacia* genome includes large, horizontally transferred genomic islands spanning up to 9.3 % of the chromosome, which are considered to be important in adaptation to different environments (Holden et al. 2009).

Bacteriophages have been proposed as effective antibacterial agents to eradicate bacterial pathogens (Hyman and Abedon 2010; Abedon 2011; Drulis-Kawa et al. 2012 and 2015). Treatment with bacteriophages allows targeting pathogens that are resistant to conventional drugs without damaging the host’s natural flora. The efficacy of bacteriophages against CF pathogens has been shown in vivo using the *Galleria mellonella* insect model and various mouse pulmonary infection models (Seed and Dennis 2009; Carmody et al. 2010; Lynch et al. 2010; Saussereau et al. 2014; Olszak et al. 2015; Danis-Włodarczyk et al. 2016). The synergistic effect of combining bacterial viruses with antibiotics may lead to higher production of phage progeny and increased phage activity, improving bacterial killing (Kamal and Dennis 2015). Phage-based treatment poses some risks and limitations; therefore, a principle requirement is phage precise characterization not only in terms of biology, but also in genomic aspects (Merril et al. 2003; Drulis-Kawa et al. 2012 and 2015). There are reports that reinforce the idea of possible temperate phage application with approaches in using them for bacterial virulence modification. The lysogenization process of temperate *Pseudomonas* phages DMS3, MP22, and D3112 caused the inhibition in the expression of virulence factors, including bacterial group motility (swarming and twitching motility) or biofilm formation, leading to significant decrease in the mortality of *Pseudomonas*-infected animals. The changes in bacterial pathogenicity after phage integration into bacterial genome can be explained by host gene disruption, CRISPR/Cas system interaction, or by mechanisms independent of the host background (Chung et al. 2012; Zegans et al. 2009; Cady et al. 2012; Drulis-Kawa et al. 2015).

To date, 37 *Burkholderia* specific phages have been deposited into GenBank (status on 28.06.2016), including members of the *Myoviridae* (18), *Siphoviridae* (9), and *Podoviridae* (10) families. The presence of a recombinase or integrase in the bacteriophage genome, indicating the temperate nature of the phage (Gill and Young 2011), was confirmed for 22 of these 37 sequenced genomes (phiE202, phi52237, KS14, KL3, KS5, BEK, and φX216 from *Myoviridae*, *Peduovirinae*, and *P2likevirus*; AH2, KL1, Bcep176, Phi1026b, phiE125, and KS9 from *Siphoviridae*; and BcepMigl, DC1, BcepC6B, BcepIL02, Bcep22 Bp-AMP1, Bp-AMP2, Bp-AMP3, and Bp-AMP4 from *Podoviridae*). Also several other viruses including *Enterobacteria* phages: P2, 186, PsP3, and Wφ; *Yersinia* phage L-413C; *Salmonella* phages Fels-2 and SopEφ; *Pseudomonas* phage φCTX; *Mannheimia* phage φ-MhaA1_PHL101; and *Ralstonia* phage RSA1 belong to the *P2likevirus* genus (Lavigne et al. 2009). Even though these phages were classified as temperate, they could be engineered as lytic mutants (Lynch et al. 2010) or provide a source for proteins (e.g., endolysins and depolymerases), which could potentially be used as enzyme-based antibacterials. Here, we report a newly discovered *B. cenocepacia* IIIA-specific temperate phage, designated AP3 (vB_BceM_AP3), and classified to the *P2likevirus* genus. This phage was characterized in terms of genome organization including lysis cassette, the integration site and lysogeny event analysis, and the tail fiber protein amino acids composition with the other P2-like phages and prophages found in host sequences. Furthermore, the efficacy of AP3 in the eradication of *B. cenocepacia* infection in moth larvae and the emergence of resistant clones was studied.

## Materials and methods

### Phage isolation and propagation

Phage AP3 was isolated from a natural wastewater treatment plant (irrigated fields) in Wroclaw (Poland) as previously described (Olszak et al. 2015). AP3 was isolated from and propagated in the clinical isolate 7780 of *B. cenocepacia* IIIA lineage (Table 1). The titer and plaque morphology were assessed by the double-agar layer technique (Adams 1959). The *B. cenocepacia* isolate 7780 has been deposited in Polish Collection of Microorganisms under accession no. PCM 2854. The phage AP3 is available in the collection of Institute of Genetics and Microbiology, Wroclaw, Poland.

**Table 1:** AP3 phage activity on the collection of BCC strains

No.	Isolate no.	BCC strains	Origin	AP3 phage 10^5 pfu/ml	AP3 phage 10^8 pfu/ml
1	5	*B. cenocepacia* lineage IIIA	Lung transplant—Freeman Hospital, Newcastle, UK	+	+
2	6	*B. cenocepacia* lineage IIIA	Lung transplant—Freeman Hospital, Newcastle, UK	+	+
3	10	*B. cenocepacia* lineage IIIA	Lung transplant—Freeman Hospital, Newcastle, UK	+	+
4	18	*B. cenocepacia* lineage IIIA	Lung transplant—Freeman Hospital, Newcastle, UK	+	+
5	19	*B. cenocepacia* lineage IIIA	Lung transplant—Freeman Hospital, Newcastle, UK	−	−
6	20	*B. cenocepacia* lineage IIIA	Lung transplant—Freeman Hospital, Newcastle, UK	+	+
7	21	*B. cenocepacia* lineage IIIA	Lung transplant—Freeman Hospital, Newcastle, UK	+	+
8	7780	*B. cenocepacia* lineage IIIA	Lung transplant—Freeman Hospital, Newcastle, UK	+	+
9	1567	*B. cenocepacia* lineage IIIA	Queen’s University of Belfast, UK	+	+
10	1571	*B. cenocepacia* lineage IIIA	Queen’s University of Belfast, UK	−	−
11	1921	*B. cenocepacia* lineage IIIA	Queen’s University of Belfast, UK	−	−
12	1945	*B. cenocepacia* lineage IIIA	Queen’s University of Belfast, UK	−	−
13	1946	*B. cenocepacia* lineage IIIA	Queen’s University of Belfast, UK	−	−
14	1947	*B. cenocepacia* lineage IIIA	Queen’s University of Belfast, UK	+	+
15	30	*B. cenocepacia* lineage IIIA	Royal Victoria Infirmary, Newcastle, UK	−	−
16	39	*B. cenocepacia* lineage IIIA	Royal Victoria Infirmary, Newcastle, UK	+	+
17	45	*B. cenocepacia* lineage IIIB	Royal Victoria Infirmary, Newcastle, UK	−	−
18	1	*B. cenocepacia* lineage IIIE	Lung transplant—Freeman Hospital, Newcastle, UK	−	−
19	35	*B. cenocepacia* lineage IIIE	Royal Victoria Infirmary, Newcastle, UK	−	−
20	14	*B. cenocepacia* lineage non-IIIA/IIIB	Lung transplant—Freeman Hospital, Newcastle, UK	−	−
21	2	*B. cepacia* group 6	Lung transplant—Freeman Hospital, Newcastle, UK	−	−
22	3830	*B. contaminans*	Queen’s University of Belfast, UK	−	−
23	3831	*B. contaminans*	Queen’s University of Belfast, UK	−	−
24	3832	*B. contaminans*	Queen’s University of Belfast, UK	−	−
25	3833	*B. contaminans*	Queen’s University of Belfast, UK	−	−
26	44	*B. contaminans*	Royal Victoria Infirmary, Newcastle, UK	−	−
27	34	*B. multivorans*	Southern Ireland, UK	−	−
28	3	*B. multivorans*	Lung transplant—Freeman Hospital, Newcastle, UK	−	−
29	4	*B. multivorans*	Lung transplant—Freeman Hospital, Newcastle, UK	−	−
30	7	*B. multivorans*	Lung transplant—Freeman Hospital, Newcastle, UK	−	−
31	9	*B. multivorans*	Lung transplant—Freeman Hospital, Newcastle, UK	−	−
32	11	*B. multivorans*	Lung transplant—Freeman Hospital, Newcastle, UK	−	−
33	12	*B. multivorans*	Lung transplant—Freeman Hospital, Newcastle, UK	−	−
34	13	*B. multivorans*	Lung transplant—Freeman Hospital, Newcastle, UK	−	−
35	17	*B. multivorans*	Lung transplant—Freeman Hospital, Newcastle, UK	−	−
36	22	*B. multivorans*	Lung transplant—Freeman Hospital, Newcastle, UK	−	−
37	23	*B. multivorans*	Lung transplant—Freeman Hospital, Newcastle, UK	−	−
38	28	*B. multivorans*	Lung transplant—Freeman Hospital, Newcastle, UK	−	−
39	31	*B. multivorans*	Lung transplant—Freeman Hospital, Newcastle, UK	−	−
40	33	*B. multivorans*	Lung transplant—Freeman Hospital, Newcastle, UK	−	−
41	38	*B. multivorans*	Lung transplant—Freeman Hospital, Newcastle, UK	−	−
42	41	*B. multivorans*	Lung transplant—Freeman Hospital, Newcastle, UK	−	−
43	24	*B. multivorans*	Lung transplant—Queensland, UK	−	−
44	25	*B. multivorans*	Lung transplant—Queensland, UK	−	−
45	1566	*B. multivorans*	Queen’s University of Belfast, UK	−	−
46	1568	*B. multivorans*	Queen’s University of Belfast, UK	−	−
47	1572	*B. multivorans*	Queen’s University of Belfast, UK	−	−
48	1573	*B. multivorans*	Queen’s University of Belfast, UK	−	−
49	1575	*B. multivorans*	Queen’s University of Belfast, UK	−	−
50	26	*B. multivorans*	Royal Victoria Infirmary, Newcastle, UK	−	−
51	27	*B. multivorans*	Royal Victoria Infirmary, Newcastle, UK	−	−
52	29	*B. multivorans*	Royal Victoria Infirmary, Newcastle, UK	−	−
53	32	*B. multivorans*	Royal Victoria Infirmary, Newcastle, UK	−	−
54	36	*B. multivorans-1*	Lung transplant—Queensland, UK	−	−
55	37	*B. multivorans-2*	Lung transplant—Queensland, UK	−	−
56	42	*B. seminalis*	Glasgow, Scotland, UK	−	−
57	40	*B. stabilis*	Southern Ireland, UK	−	−
58	43	*B. stabilis*	Lung transplant—Freeman Hospital, Newcastle, UK	−	−
59	8	*B. vietnamiensis*	Lung transplant—Freeman Hospital, Newcastle, UK	−	−
60	15	*B. vietnamiensis*	Lung transplant—Freeman Hospital, Newcastle, UK	−	−
61	16	*B. vietnamiensis*	Lung transplant—Freeman Hospital, Newcastle, UK	−	−

*+* active, *−* no activity

### Transmission electron microscopy (TEM)

A filtered high-titer phage lysate was centrifuged at 25,000×*g* for 60 min. The pellet was washed twice in ammonium acetate (0.1 M, pH 7.0). Phages were deposited on copper grids with carbon-coated Formvar films (Sigma-Aldrich Co., St. Louis, MO, USA) and stained for 10 s with uranyl acetate (2 %, pH 4.5). Excess liquid was blotted off and phages were examined using a Zeiss EM 900 electron microscope (Carl Zeiss Microscopy GmbH, Jena, Germany) at the Laboratory of Microscopy Techniques, University of Wroclaw, Poland. The magnification was calibrated using T4 phage tail length (114 nm) as a standard.

### DNA isolation, purification, and sequencing

Phage DNA isolation was performed with the modified protocol for λ DNA isolation (Sambrook and Russell 2001) from purified phage particles (10^8^ pfu/ml). The phage AP3 genome was sequenced using an Illumina MiSeq platform available at the Department of Applied Sciences, University of Northumbria (UK). A quality filtering, assembly, and draft genome was applied using CLC genomics workbench v. 7.5 (CLC Bio, Aarhus, Denmark). Low quality reads (<0.05), reads with two or more ambiguities, and reads shorter than 100 bp were removed. After trimming and filtering, there were 476,529,248 bp from 2,042,560 reads with an average length of 233.3 bp. The processed reads were used for reference genome assembly with *Burkholderia* phage KS5 (GenBank accession number GU911303) used as reference and de novo assembly, yielding 2016 contigs with an average contig size of 3836 bp; the N50 contig size was 6333 bp with the largest contig 16,375 bp. A high-quality draft genome was assembled by closing the gaps between the scaffolded contigs of the reference assembly by contig stitching with de novo contigs. The final draft genome comprised one scaffold, counting for a length of 36,499 bp, with an average coverage of 5639-fold. The draft genome has an average GC content of 64.04 %.

### Genome annotation and in silico analysis

The start of the AP3 genome was inferred by a location of the left *cos* site, while the genes were oriented based on the comparison to the type phage of the *P2likevirus* (*Enterobacteria* phage P2, GenBank accession number: NC_001895) and three other similar P2-like phages found in the NCBI database: φE202 (GenBank accession number: CP000623), φX216 (GenBank accession number: JX681814), and φ52237 (GenBank accession number: NC_007145). Even though three other P2-like *Burkholderia* phages were used in further analysis, only φE202, φX216, and φ52237 start with the portal vertex protein, as the type species P2-like *Enterobacteria* phage P2, which was used as the main genome template. Open reading frames (ORFs) were predicted with GeneMarkS and verified in following steps of annotation (Besemer et al. 2001). The annotation of the AP3 genome was performed using Artemis (Rutherford et al. 2000). Protein domains and the search for proteins with a similar structure were investigated with HMMER (Finn et al. 2011). ORFs were compared with existing data stored in the NCBI database, using BLASTN and BLASTP (Altschul et al. 1990; Gish and States 1993). Further, TBLASTX was used to search for conserved protein coding sequences, not previously annotated. Proteins with the highest BLAST bit score were noted for each open reading frame, along with available known domains. Search for possible transfer RNAs was done using tRNAscan-SE and Aragorn (Schattner et al. 2005; Laslett and Cänback 2004). Putative Rho-independent terminators were investigated with ARNold (Gautheret and Lambert 2001). The 100-nt sequences upstream from each putative ORF were extracted via STORM to create input data for repetitive motifs identification (Lavigne et al. 2003). MEME was used to find conserved motifs in the genome (Bailey et al. 2009) followed by manual verification of each motif. The AP3 genome organization was visualized in Circos (v. 0.67, Canada’s Michael Smith Genome Sciences Centre). The genome of bacteriophage AP3 was deposited in the GenBank under accession number KP966108.

To confirm the taxonomic cluster of AP3 as a novel phage, nucleotide comparison of genomes of different species among *P2likevirus* genus were made with CoreGenes 3.5 (Turner et al. 2013) using a gene similarity setting to at least 80 %. Phages used for comparison included BCC viruses, which query cover identity was at least 85 %—KS5 (accession: GU911303), KL3 (accession: GU911304), φE202 (accession: CP000623), BEK (accession: CP008753), φX216 (accession: JX681814), and φ52237 (accession DQ087285). Data for the linear genome visualization was acquired using TBLASTX (Altschul et al. 1990) and included in EasyFig 2.1 (Sullivan et al. 2011).

Identification and in silico analysis of the lysis proteins and lysis cassette in the AP3 genome and selected BCC phages was performed by protein and nucleotide BLAST analysis (Altschul et al. 1990), HMMER (Finn et al. 2011), HMMTOP, TMHMM (Bernsel et al. 2009), and the ExPASy SIB Bioinformatics Resource Portal (Krogh et al. 2001). The amino acid sequence similarity of individual lysis proteins between AP3, *Enterobacteria* P2, and selected BCC P2-like viruses was prepared in Circos (v. 0.67, Canada’s Michael Smith Genome Sciences Centre).

### Burst size experiments and sensitivity of phage particles to temperature, chloroform, and pH

A one-step growth curve of the isolated *Burkholderia* phage was performed as described (Pajunen et al. 2000) with some modifications (Kesik-Szeloch et al. 2013). The sensitivity of phage particles to temperature, chloroform, and pH was evaluated as previously described (Eriksson et al. 2015). Purified viruses (Szermer-Olearnik and Boratyński 2015) were suspended in phosphate-buffered saline (PBS). An equal volume of filter-sterilized bacteriophage containing 10^8^ plaque-forming units per milliliter (pfu/ml) was mixed with chloroform and incubated for 2 and 24 h at room temperature (RT), with intermittent shaking. To establish the visions’ stability at different pH values, a phage suspension of 10^8^ pfu/ml was diluted 10-fold in citric acid-Na_2_HPO_4_ buffer solution at pH 2, 4, 5, 6, and 8 and sodium carbonate-sodium bicarbonate buffer solutions at pH 10 and incubated for 1 or 5 h at RT. Phage samples were also incubated at 60, 70, and 80 °C for 10 and 30 min. Phage titers were assessed using the soft agar overlay method (Adams 1959; Kutter 2009). Phage particles in PBS (pH 7.4) were used as a control in all the previously described tests.

### Determination of host range

The host specificity of AP3 phage was determined by spot testing applying 10^5^ and 10^8^ pfu/ml (Kutter 2009) on 61 BCC clinical isolates (Table 1). Type IIIA and IIIB lineages among *B. cenocepacia* strains were identified by PCR (Mahenthiralingam et al. 2000). Sense and antisense primers 5′-GCTCGACGTTCAATATGCC-3′ and 5′-TCGAGACGCACCGACGAG-3′ were used for IIIA detection (annealing temperature 62 °C; expected product size 378 bp) and primers 5′-GCTGCAAGTCATCGCTGAA-3′ and 5′-TACGCCATCGGGCATGCT-3′ were used for IIIB detection (annealing temperature 60 °C; expected product size 781 bp). Pulsed-field gel electrophoresis (PFGE) was employed to discriminate the clonal origin of *B. cenocepacia* IIIA isolates sensitive to AP3 infection. PFGE was carried out according to Chua et al. (2011) using a one-step lysis procedure in which bacterial cells, immobilized in an agarose gel, were lysed in lysis buffer containing 50 mM Tris, 50 mM EDTA, 1 % SDS, and 100 μg/ml proteinase K (pH 8.0) at 56 °C for 2 h. Next, agarose plugs were thoroughly rinsed with TE buffer (10 mM Tris, 1 mM EDTA, pH 8.0) and digested with *Xba*I (Thermo Fisher Scientific, Fermentas Life Science, Waltham, MA, USA). DNA fragments were separated by PFGE on a CHEF DRII apparatus (Bio-Rad Laboratories, Hemel Hempstead, UK) in a 1 % agarose gel in 0.5 × TBE buffer at 12 °C. A linear ramp of 5 to 65 s was used, and gels were run for 20 h at 200 V. MidRange PFGE Marker (New England Biolabs) was used as a mass marker. DNA was stained with ethidium bromide. Bacteria were stored at −70 °C in trypticase soy broth (TSB; Becton Dickinson and Company, Cockeysville, MD, USA) supplemented with 20 % glycerol (Avantor Performance Materials Poland S.A., Gliwice, Poland).

### The emergence of phage-resistant clones

The *B. cenocepacia* 7780 host strain was cultured in 5 ml of TSB (Becton Dickinson and Company, Cockeysville, MD, USA) at 37 °C for 3 h to 10^8^ cfu/ml. One hundred microliters of AP3 phage (10^7^ pfu/ml) was added to the bacterial culture and incubated at 37 °C during 24 h. The surviving bacterial cells were obtained by plating on TSA plates (Becton Dickinson and Company, Cockeysville, MD, USA). Twenty randomly isolated colonies were picked for further analysis and examined for AP3 sensitivity by spot testing, applying either 10^7^ or 10^5^ pfu/ml to visualize individual plaques (Kutter 2009). BCC resistance to phage infection was considered as possible lysogeny by AP3 temperate phage during the bacterial culture infection. The presence of the AP3 genome within the bacterial cell was confirmed by PCR according to Kvitko et al. (2012). First, the unique AP3 gene vB_BceM_AP3_0002 encoding cytotoxic translational repressor of toxin-antitoxin stability system was chosen for confirmation of AP3 genome presence inside the host cell (F: 5′-ATGAACTCGATCAAATGGACCCC-3′; R: 5′-TTAGTACGTGCGTTCATCGCGTTTC-3′; annealing temperature 57 °C; expected product size 254 bp). Second, the phage integration next to the bacterial tRNA gene was determined to match *attR* and *attL* sites, respectively (F: 5′-GCGCCCGTAGCTCAATGG-3′; R: 5′-TCAGACGTCGAGCTGCGCG-3′; annealing temperature 57 °C; expected product size 806 bp). The episome form of AP3 was confirmed by the detection of the intact integrase gene vB_BceM_AP3_0051 (F: 5′-ATGTCGCTCTATAAACGCAGTAACAGTCCC-3′; R: 5′-TCAGCCGGCGGCGACGTGCAG-3′; annealing temperature 57 °C; expected product size 1076 bp).

### *G. mellonella* larvae infection model

The *G. mellonella* infection model, performed as described by Cullen et al. (2015), was used to assess the virulence of selected isolates. Briefly, fresh bacterial cultures in LB broth were diluted serially in saline solution to densities ranging between 10^6^ and 10^8^ cfu/ml. Each dilution was injected (10 μl) into the hindmost proleg of healthy larvae (20 per group). Bacterial burden was determined by plating 10 μl of the serial dilutions onto LB agar and colonies were counted after 24 h. The injected larvae were incubated at 37 °C for 24 h; the numbers of dead larvae were counted and the LD_50_ values determined and presented as a mean (*N* = 3 experiments). The in vivo phage treatment assay was conducted on a wax moth larvae model as described previously (Olszak et al. 2015). To assess the antibacterial activity of AP3, larvae (*N* = 60) were sequentially injected with a 10-μl lethal dose of 10^5^ cells of *B. cenocepacia* 7780 host strain and 10 μl of phage lysate at the titration equal to multiplicity of infection (MOI) 10. After injection into the ventral side, the larvae were incubated for 72 h at 37 °C and the results were expressed as the percentage survival rate assessed by the lack of touch-provoked motility and the appearance of pigmentation at 18, 24, 36, 48, and 72 h post-injection. Experiments were performed six times (10 larvae per trial). The controls consisted of uninfected and larvae receiving phage lysate only or saline solution (negative) and infected with bacterial lethal dose (positive; *N* = 10 per each probe). The analysis of survival curves was performed by the log-rank Mantel–Cox test using GraphPad Prism software (GraphPad Software, Inc., La Jolla, USA). *P* values <0.05 were considered statistically significant.

## Results

### Phage isolation and morphology

**Fig. 1:**
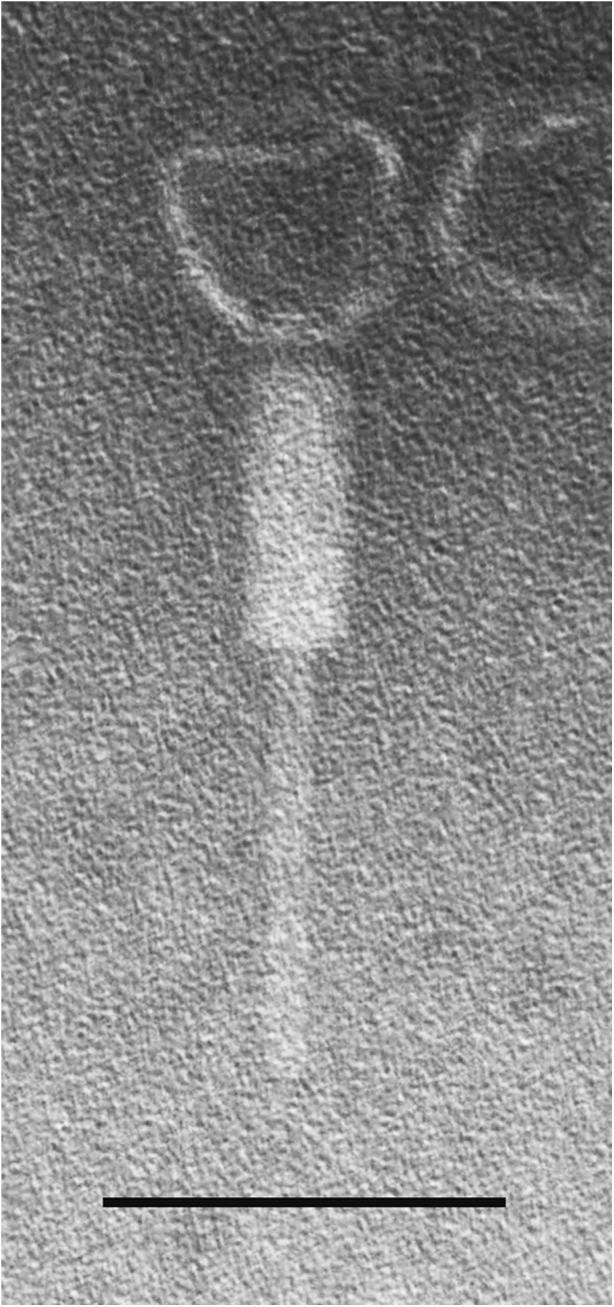
Transmission electron micrograph of AP3. The sample was negatively stained with 2 % uranyl acetate and viewed at 80,000-fold magnification. The *size bar* is 100 nm

Phage AP3 was isolated in a water sample from irrigated fields and propagated on the clinical *B. cenocepacia* 7780 strain, examined by TEM, and morphologically classified as a Caudovirales, Myoviridae member of the Peduovirinae subfamily (Fig. 1). Its icosahedral head was estimated to be approximately 64 nm between opposite apices and connected to a long-contractile 175-nm tail. The closely related phages KS5, KS14, and KL3 have similarly sized capsids, each 65 nm in diameter; but in contrast, their tails vary in length, 140 nm for KS14, 150 nm for KS5, and 160 nm for KL3 (Lynch et al. 2010).

### Genome analysis

The AP3 genome is 36,499 bp with a G + C content of 64.04 %. Nucleotide sequence comparison revealed that AP3 is predicted to possess a 55-bp cohesive (*cos*) region, conserved between the known *cos* regions of P2-like phages (Supplementary Fig. S1). *Cos* regions in P2 and related phages determine genome ends and are essential for transduction and phage packaging (Ziermann and Calendar 1990). As for P2-like *Pseudomonas* phage phiCTX (NC_003278.1), the *cos* region of AP3 is in close proximity to its integrase, which is a unique position. However, similarly to all of P2-related phages, within the *cos* region of AP3, GC-rich 20-nt 5′-extruding cohesive ends (5′ GGTGGGGCGGGGTCACAACT) can be predicted. Analogous to the P2-related phages, the *cos* site was set as the start (*cosL*; left cohesive end) and end (*cosR*; right cohesive end) of the AP3 genome.

**Fig. 2:**
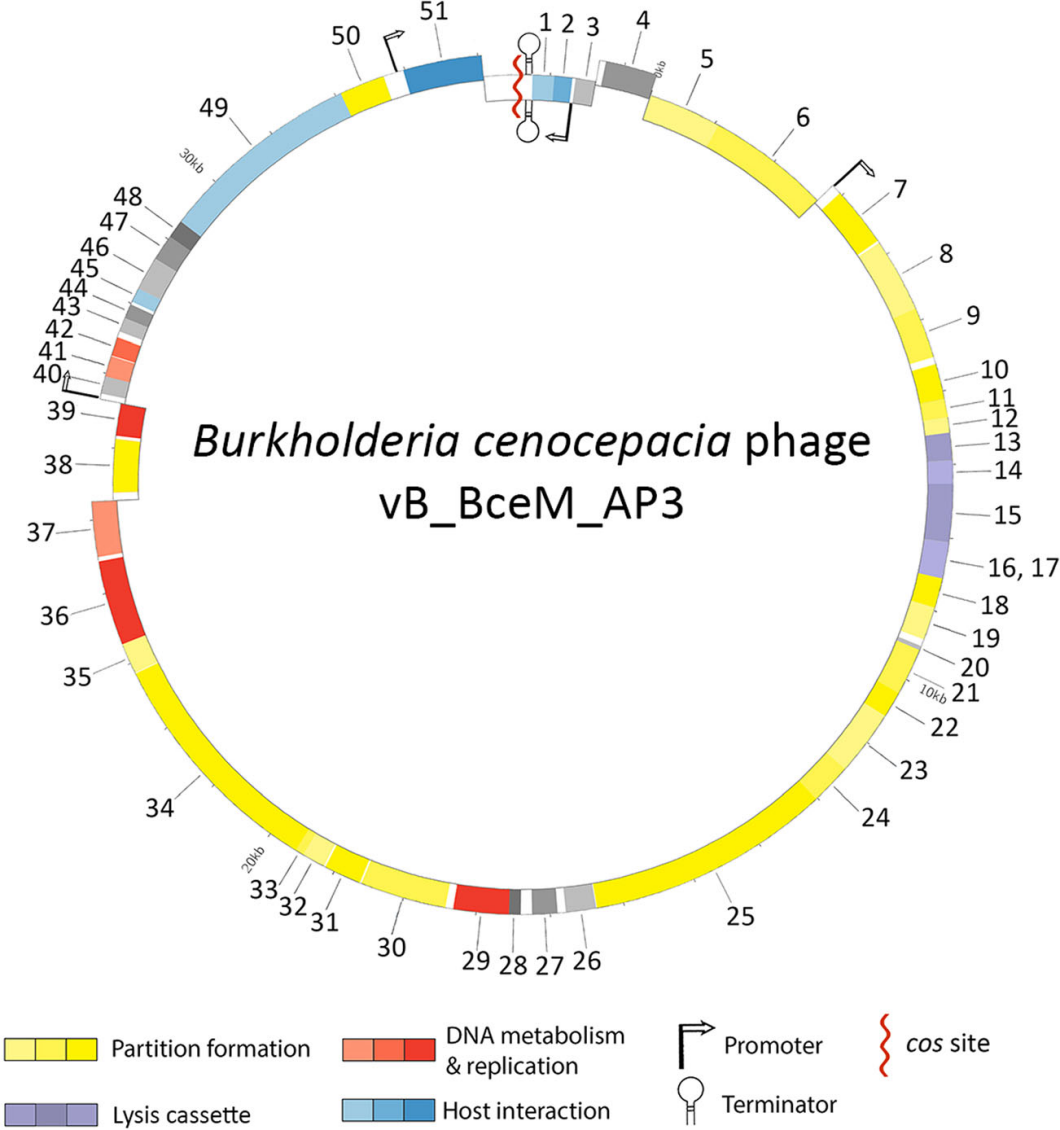
Genome map of *Burkholderia* phage AP3. Structural protein genes (*yellow*), lysis cassette (*green*), DNA metabolism and replication (*red*), host interaction protein genes (*blue*), and hypothetical protein genes (*gray*). Both terminators and promoters are also noted on the map

There are 51 predicted CDSs: 44 start with ATG codon (Met), 6 start with GTG (Val), and 1 start with TTG (Leu). The circular map of AP3 phage genome organization shows clearly defined genome organization, with host interaction, DNA metabolism and replication, structural, and lysis and lysogeny coding genes (Fig. 2). This last region of genes also comprises an interesting cytotoxic translational repressor of toxin-antitoxin stability, which may be involved in an abortive phage infection system (Labrie et al. 2010). Supplementary Table S1 shows key annotation information including locus tag, molecular data, and homologs with predicted protein motifs for all proteins. Also, the annotation uses the gene numbers corresponding to those assigned the Circos map (Fig. 2). Four host promoters located in the intergenic regions of AP3 genome were predicted based on analysis of recurrent motifs in the intergenic regions (Supplementary Table S2). Given their specific location, it is likely that they cover transcription of early, middle, and late genes. No conserved phage promoter motifs could be identified during the analysis. A single factor-independent terminator was predicted with transcription termination functions at the negative and positive strands. No tRNA-coding sequence could be discerned in the AP3 genome.

Based on BLASTN, the AP3 nucleotide sequence shows high homology to six other P2-like phages (Supplementary Table S3). The related phage genome lengths vary between 35,741 (φE202) and 40,555 bp (KL3), as does the G + C content, ranging from 63.22 (KL3) to 68.82 % (BEK). The number of protein coding sequences ranges between 45 (KS5) and 57 (BEK). An interesting aspect, which was not noted previously, is the distribution of the CDSs. Even though there are only relatively small differences in the lengths of the genomes, significant differences exist in the number of coding sequences. The most similar phage to AP3 was KS5, which infects *B. cenocepacia* (Lynch et al. 2010). Phage KL3, which has the largest genome among all compared phages, has only two CDSs more than AP3, while the ∼3 kbp smaller phage BEK has seven more. Based on the CoreGenes 3.5 output table, all phages share 31 homologous genes, 8 of which are hypothetical and 23 are genes encoding proteins with a predicted function.

**Fig. 3:**
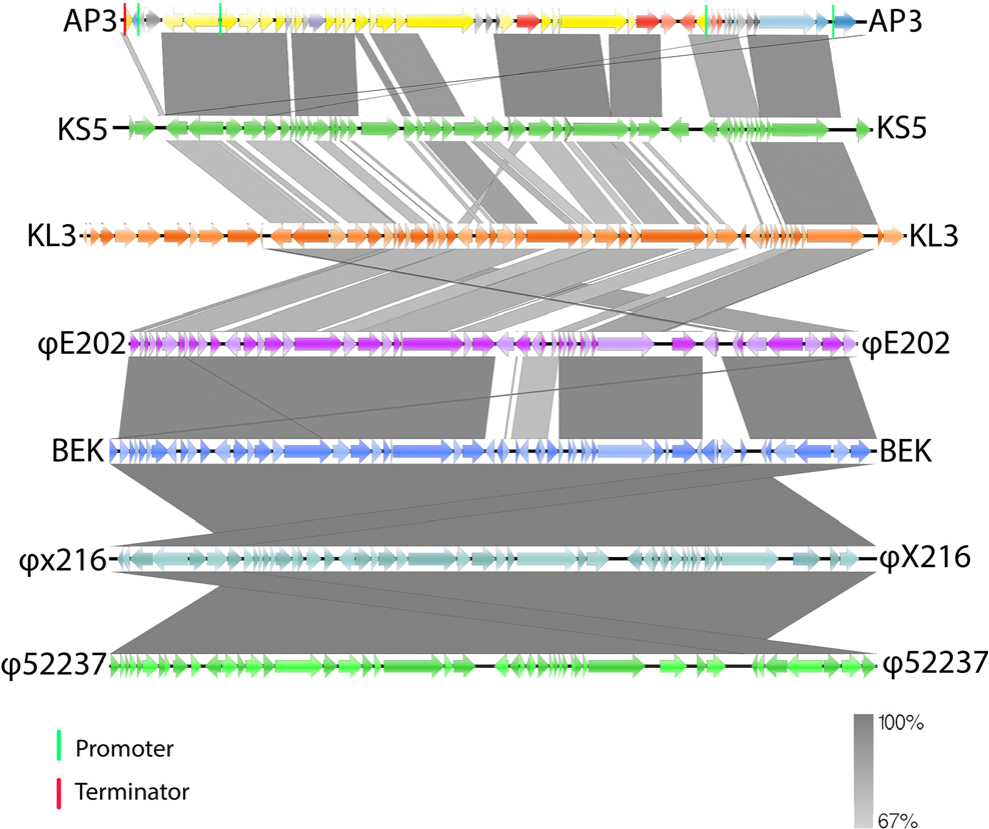
Pairwise comparison using TBLASTX of the AP3 and six selected BCC P2-like phages. *Lines* linking genomes represent high pairwise similarity between phages. Putative phage specific promoter sites (*green lines*), rho-independent terminator (*red lines*), and percentage of similarity between similar regions (*shade scale* on the *bottom* of the figure)

Figure 3 shows the genes of AP3 compared to other P2-like phages along with the direction of their transcription. Genomes were arranged with the most similar phages lying adjacent to AP3, while the least similar phages are more distant. The comparison suggests that KS5 holds closest relation to AP3 and φ52237 is least homologous. The KL3, φE202, φX216, and φ52237 phages show a high level of nucleotide similarity among each other but less to KS5 and AP3. Further genome analysis indicated that despite BLASTN showing significant similarity between seven selected BCC phages, the annotation differed. Starting positions of AP3 vary for closely related phages available in the database. In KS5, KL3, and φ52237, all genes were annotated on the opposite strand in comparison to AP3 genome. Genomes of BEK, φE202, and φX216 are oriented according to the *Enterobacteria* phage P2. None of the selected phages have predicted promoter and terminator sequences in their GenBank files and the comparison of those elements was omitted in our analysis.

### Lysis cassette

**Fig. 4:**
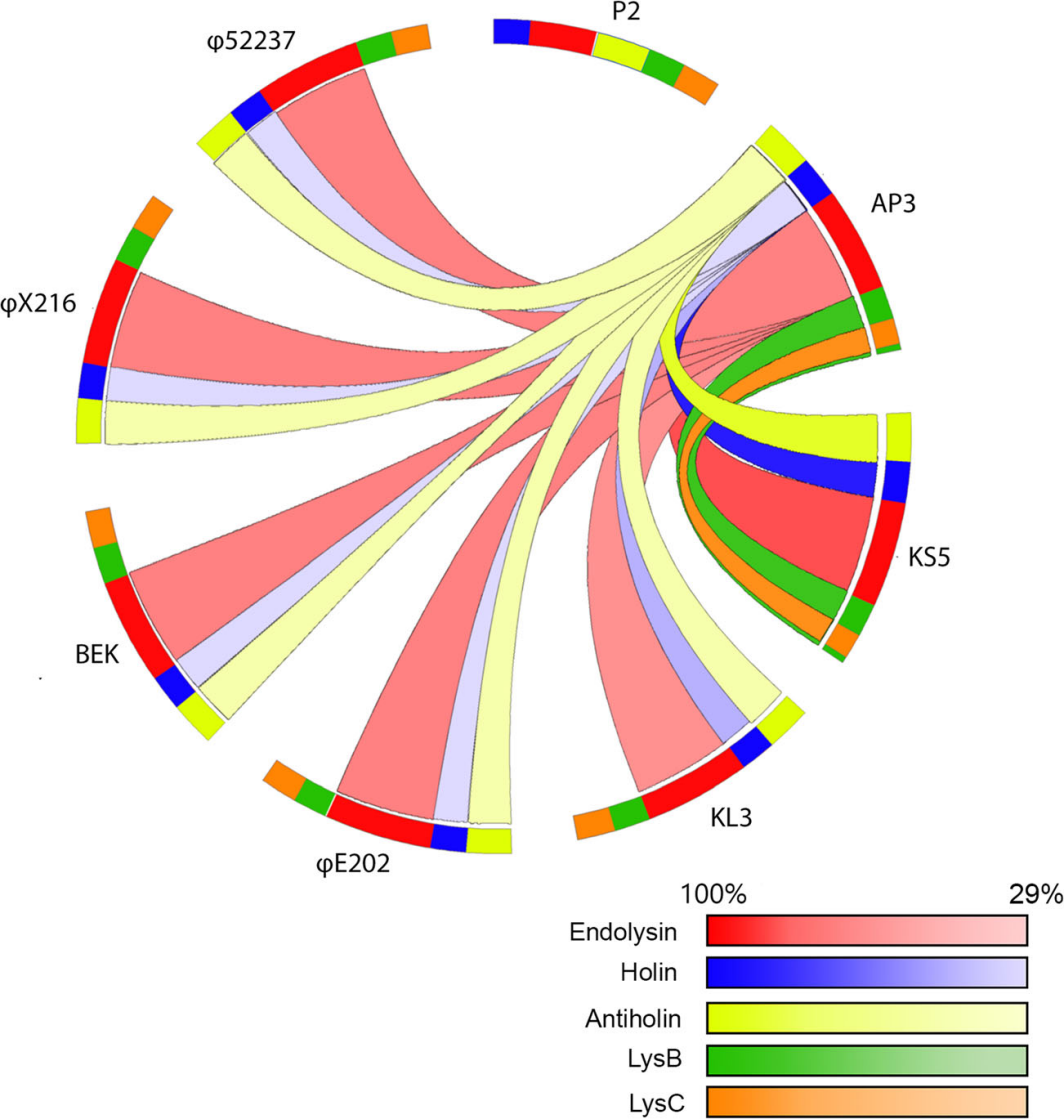
The organization of the lysis cassette of *Enterobacteria* P2 and selected BCC P2-like viruses

A closer inspection of the AP3 genome reveals a four-component lysis cassette comprising a putative antiholin, putative holin, endolysin, and spanins, similar to the model described in detail for λ, T4, and P2 phages (Berry et al. 2013; Garrett et al. 1981; Morita et al. 2001; Moussa et al. 2012; Summer et al. 2007a; To et al. 2013; White et al. 2011; Young 2014). In this model, the holin perforates the inner membrane and forms non-specific channels required for the transport of endolysin across the membrane. In AP3, the predicted holin (AP3_0014) is a small-size protein (106 residues; 11.3 kDa) with two hydrophobic transmembrane helices, classified as a class II holin. The phage holin activity is regulated by the antiholin, which prevents premature lysis (Ryan and Rutenberg 2007). The predicted antiholin of AP3 (AP3_0013) is also a small protein (124 residues; 12.7 kDa) that possesses two transmembrane domains. In the periplasmic space, the released endolysin destroys the peptidoglycan bonds, leading to host lysis by the internal turgor pressure. The AP3 endolysin (AP3_0015) is a modular protein (266 residues; 28.9 kDa) with two conserved domains: a putative peptidoglycan binding domain PG_binding_1 and a DUF3380 domain, which was recently associated with a phage-mediated N-acetylmuramidase activity of a *Salmonella* phage (Rodríguez-Rubio et al. 2016). The last part of the system comprises a spanin (unimolecular or as a complex). Spanin is anchored to both the inner and outer cell membranes and causes the physical destruction of the outer membrane. In AP3, the spanin moiety of the lysis cassette is predicted to be a complex of two proteins i-spanin and o-spanin encoded by separate genes: AP3_0016 and AP3_0017, respectively. The AP3_0012 product is a predicted integral membrane protein (162 residues, 17.1 kDa) with one N-terminal transmembrane domain. The AP3_0013 gene, encoding an outer membrane lipoprotein (66 residues, 7.0 kDa), overlaps the +1 frame within the i-spanin gene. A comparative analysis of the lysis cassette was also performed among AP3, the closely related phages KS5, KL3, φE202 φX216, BEK, and φ52237, and the reference *Enterobacteria* P2 phage (Fig. 4). This analysis demonstrated a closer relationship in the lysis cassettes of KL3, φE202 φX216, BEK, and φ52237 phages than in those of AP3 and KS5, which are more similar to each other.

### Microbiological analysis of *Burkholderia* phage AP3

AP3 forms small, clear plaques of about 1 mm diameter when tested with the soft agar overlay method. In one-step growth curves, the latent period was estimated around 60–65 min, and approximately 40–45 progeny virions are produced per infected host cell. The bacteriophage retains almost 100 % infectivity after incubation at pH 4–10 up to 5 h, whereas pH 2 reduces the phage titer completely after only 1 h of incubation. Ten minutes exposure to 60 °C halves the pfu number, and after 30 min, a 10-fold reduction was observed, while the incubation for 10 min at 70 °C results in a decrease of five orders of magnitude in phage titer. The AP3 virions are completely destroyed after 30 and 10 min incubation at 70 and 80 °C, respectively. Stability tests show that AP3 is extremely sensitive to chloroform with a four-order reduction in phage titer after 2 h exposure and no remaining infective particles after 24 h. This is consistent with some reports about chloroform-sensitive tailed phages examined by our group and other researchers earlier (Kesik-Szeloch et al. 2013; Ackermann 2006). Sixty-one BCC representative strains (Table 1) were used for the AP3 host range assays: *B. cenocepacia* (20 isolates), *B. cepacia* (1 isolate), *B. contaminans* (5 isolates), *B. multivorans* (29 isolates), *B. seminalis* (1 isolate), *B. stabilis* (2 isolates), and *B*. *vietnamiensis* (3 isolates). AP3 was only able to propagate on 10 of the 16 *B. cenocepacia* IIIA isolates. PFGE analysis reveals differences in restriction patterns for each *B. cenocepacia* IIIA isolate, indicating that the sensitive bacteria do not belong to the same clone (Supplementary Fig. S2). As the specific recognition of bacterial receptor is the main step of successful phage adsorption, a detailed in silico analysis of the tail fiber protein (TFP) was performed. Phages infecting different species and sharing high homology of tail fiber proteins are successful in cross-infecting their host strains (Smith et al. 2007; Simpson et al. 2015). Phage tail fiber protein (vB_BceM_AP3_0025) from phage AP3 was compared to the NCBI protein database using BLASTP to find its homologs occurring in different species. Eight hits holding similarity of more than 70 % were investigated further. One sequence belongs to *Burkholderia* phage ST79 (lytic phage), while all other proteins are part of temperate bacteriophages genomes, integrated in bacterial genomes. These strains include *B. cenocepacia* D2AES, *B. cenocepacia* PT15, *B. glumae* 3252-8, *B. gladioli* UCD-UG CHAPALOTE, *B. glumae* NCPPB 3923, *B. glumae* BGR1, and *B. glumae* LMG 2196. Database records of these genomes (except LMG 2196) have been generated using whole genome shotgun (WGS) techniques and were not assembled into one complete scaffold. Genes found in all eight strains hold strong similarity in amino acid content (Supplementary Fig. S3). The origin, location, and size of the homologous genes, as well as precise level of their similarity (ranging between 72 and 82 %), are presented in Supplementary Table S4. From all inspected genomes, *B. glumae* BGR1 tail fiber protein shows the lowest homology to AP3 phage, whereas *B. cenocepacia* D2AES was most similar. The latter gene product also has the approximate number of amino acids (1114 aa) as the AP3 phage (1113 aa). As expected, the highest homology scores could be observed among temperate phages whose hosts belong to *B. cenocepacia* group. Similar comparison of tail fiber protein was conducted between phage AP3 and closely related *Burkholderia* phages KS5, KL3, φE202, BEK, φX216, and φ52237 (Supplementary Fig. S4). The comparison via ClustalOmega (Goujon et al. 2010; Sievers et al. 2011) shows high similarity on the N-terminus part of TFP which serves as connector to the phage structural proteins and dropping significantly while moving to the C-terminus direction which is responsible for host receptor recognition and binding (Simpson et al. 2015). Interestingly, the AP3 phage TFP is twice as long as any other protein compared, having additional 365 aa at C-terminal end. A smaller difference in length might be observed between AP3 and KL3 phage, as there is an additional 97 aa region for KL3. All phages are highly similar on the N-terminal ∼170 aa, after which similarity level drops. At the C-terminal end (after 740 aa), AP3 phage TFP is unique and does not resemble any of the compared bacteria or phages.

### *Galleria* infection treatment assay and the emergence of phage-resistant mutants

**Fig. 5:**
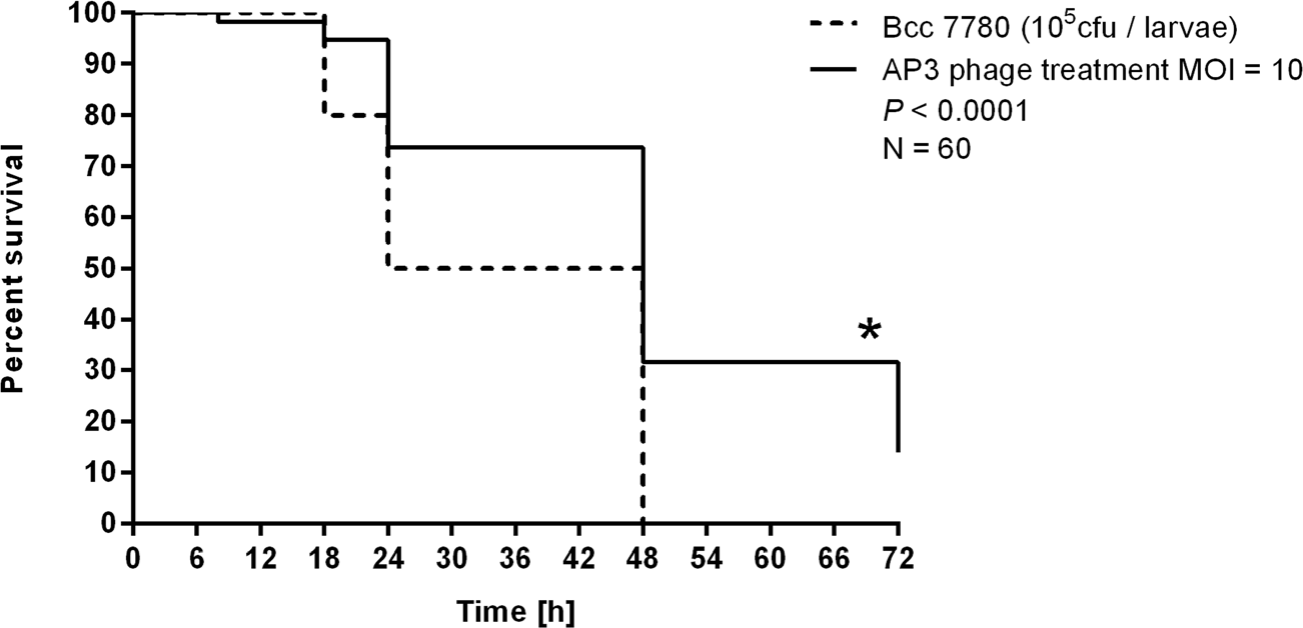
Antibacterial activity of AP3 phage (MOI 10) in the treatment of *B. cenocepacia* 7780-infected *Galleria* larvae. Statistical analysis was calculated for pairwise comparisons between infected larvae and phage-treated infected larvae using Mantel–Cox test (denoted *P* values <0.05).

The wax moth larvae model was used to evaluate in vivo the antibacterial efficacy of AP3 temperate phage in the eradication of *B. cenocepacia* 7780 infection. Larvae were inoculated with the LD_50_ dose of 10^5^ cells per larvae followed by the injection of AP3 phage within 30 min post-infection at the MOI of 10 (Fig. 5). The negative controls (uninfected larvae and larvae phage lysate treated; *N* = 30 each probe) show a 100 % survival rate. The survival of BCC infected larvae was 80, 60, and 0 % after 18, 24, and 48 h post-injection, respectively. In contrast, AP3 phage application significantly extends larvae survival (*P* < 0.0001) to 95, 72, 30, and 15 % surviving larvae after 18, 24, 48, and 72 h post-infection, respectively.

**Fig. 6:**
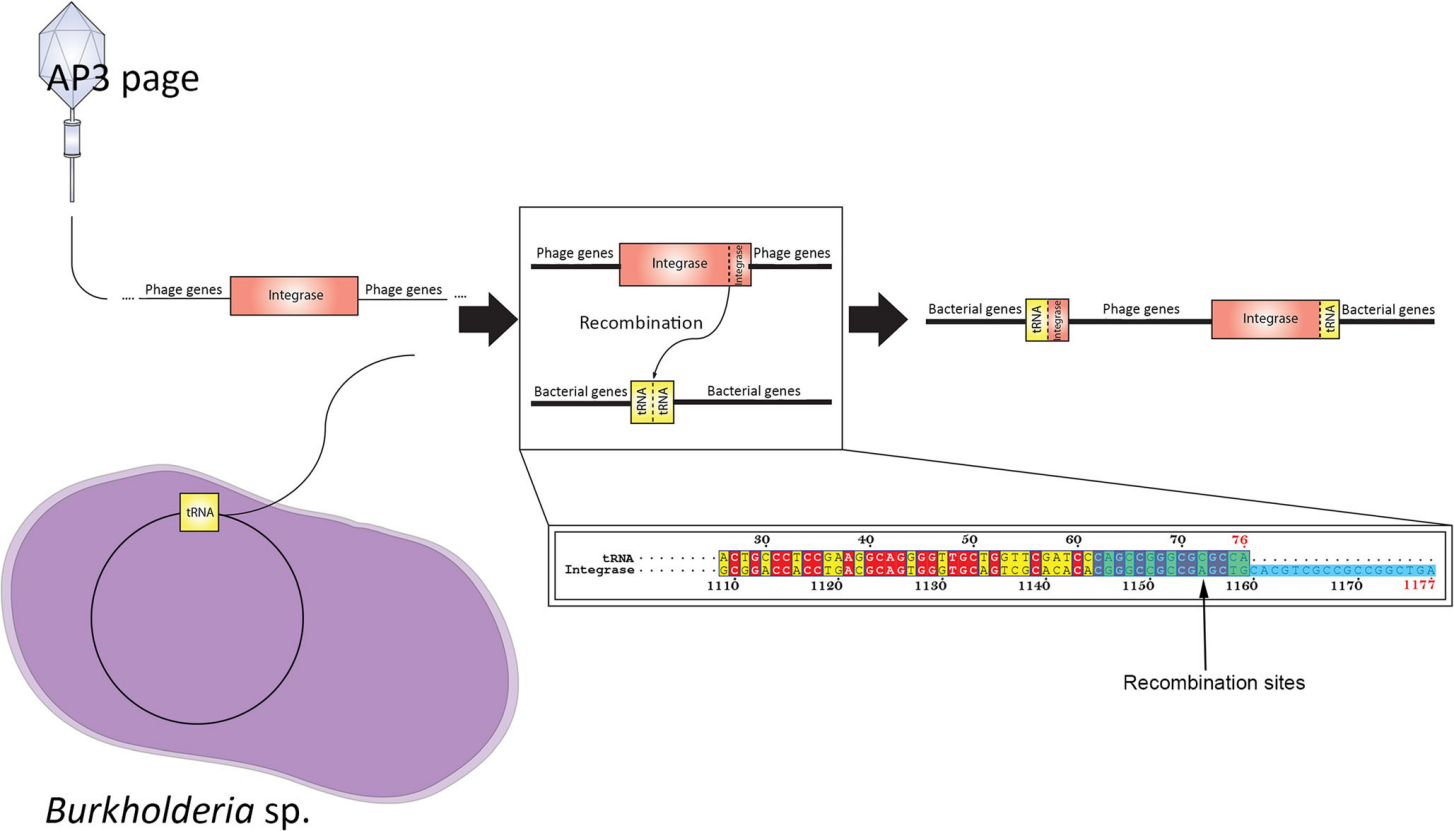
The scheme of AP3 phage integration site

Phage treatment results in the emergence of phage-resistant variants. We therefore analyzed the emergence of AP3-resistant variants at 24 h after infection in broth culture (Table 2). From 20 randomly chosen colonies, four (M1-3 to M1-6) remain susceptible to AP3 phage infection, while the rest of the 16 clones became resistant to AP3. PCR analysis was used to assess whether the resistance depends on the integration of the AP3 genome in the bacterial chromosome. The integration site of AP3 was initially based on sequence similarity to other P2-like *Burkholderia* phages which encode integrase and was confirmed experimentally. Two integration sites were previously described for BCC phages—tRNA genes and AMP nucleosidases (Lynch et al. 2010; Ronnig et al. 2010). The latter one was thought to be less probable, due to the high similarity of AP3 to KL3, φ52237, and φE202 viruses, which all integrate in tRNA genes (either tRNA[Thr] or tRNA[Phe]). This was further confirmed using PHAST (Zhou et al. 2011), which predicts a similar phage integrated at the end of a *B. cenocepacia* MC0-3 chromosome 1 (CP000958) tRNA, with the *attP* site in downstream of the integrase. During the recombination of *attP* and *attB*, the integrase splits into two fragments substituting last 33 nucleotides (located on its 3′ end) with last 15 nucleotides of bacterial tRNA-Arg (also located on 3′ end; Fig. 6). This process does not influence the functionality of the bacterial tRNA-Arg, which is produced even after phage integration. The acquisition of AP3 phage was confirmed by the presence of a gene encoding a translational repressor of a toxin-antitoxin stability system. All 16 resistant bacterial variants were positive for this unique AP3 gene and similarly for the intact integrase gene, indicating the episomal state of the phage. The phage integration site next to the bacterial tRNA gene was confirmed for 10 of the 16 isolates (Table 2). All resistant variants were as virulent as the parental strain in the *Galleria*, indicating that AP3 lysogeny had no significant effect in *B. cenocepacia* pathogenicity.

**Table 2:** *B. cenocepacia* 7780 variants isolated from the culture after AP3 phage propagation

No.	Bacteria	AP3 phage activity		Unique gene presence	Integrase gene presence	Integration next to tRNA gene	LD_50_ per *Galleria* larvae [*N* = 20]
		10^5 pfu/ml	10^7 pfu/ml				
1	Wild type 7780	+	+	−	−	−	10^5
2	M1-1	−	−	+	+	−	10^5
3	M1-2	−	−	+	+	−	10^5
4	M1-3	+	+	−	−	−	10^5
5	M1-4	+	+	−	−	−	10^5
6	M1-5	+	+	−	−	−	10^5
7	M1-6	+	+	−	−	−	10^5
8	M1-7	−	−	+	+	−	10^5
9	M1-8	−	−	+	+	+	10^5
10	M1-9	−	−	+	+	−	10^5
11	M1-10	−	−	+	+	+	10^5
12	M2-6	−	−	+	+	+	10^5
13	M2-7	−	−	+	+	−	10^5
14	M2-8	−	−	+	+	+	10^5
15	M2-9	−	−	+	+	+	10^5
16	M2-10	−	−	+	+	−	10^5
17	M3-6	−	−	+	+	+	10^5
18	M3-7	−	−	+	+	+	10^5
19	M3-8	−	−	+	+	+	10^5
20	M3-9	−	−	+	+	+	10^5
21	M3-10	−	−	+	+	+	10^5

*+* active, *−* no activity, *M* isolated clone after phage treatment

## Discussion

In this manuscript, we report the isolation and characterization of the *Burkholderia* phage vB_BceM_AP3 (AP3), a member of the *P2likevirus* genus, as a potential antibacterial agent. AP3 exhibits high pairwise nucleotide identity (61.7 %) to another *B. cenocepacia* infecting phage KS5 and lower homology (46.7–49.5 %) to the remaining *P2likevirus* phages specific to *Burkholderia* species. The similarities between AP3 and compared temperate phages (KL3, φ52237, and φE202) can be seen in terms of integration site found as tRNA (either tRNA[Thr] or tRNA[Phe]; Zhou et al. 2011), with the *attP* site downstream of phage integrase sequence homologous to the end of bacterial tRNA gene; thus, host gene disruption is unlikely (Fig. 6). Further comparison of *P2likevirus* BCC phages reveals a conserved organization of the lysis cassette (putative antiholin, putative holin, endolysin, and spanins) and show a >86 % homology between AP3 and KS5 specific lysis genes. Therefore, we conclude these temperate phages share a conserved lysogenization and lysis strategy. Considering phage application as an antimicrobial agent, it is important to choose phages with a wide host range for more effective use against different clinical isolates (Abedon 2011; Drulis-Kawa et al. 2012). The crucial step of successful phage infection is the ability of specific host receptor recognition conditioned mostly by TFP. The AP3 temperate phage described here is specific to *B. cenocepacia* lineage IIIA strains and can propagate on 10 out of 18 multidrug-resistant clinical strains. The TFP amino acids sequence comparison revealed that AP3 phage probably recognizes another bacterial receptor than the other *P2likevirus* representatives and it is the first isolated and sequenced *Burkholderia* phage from this genus highly similar to prophages found in *Burkholderia* sp. genomes.

AP3 was very effective to eradicate *B. cenocepacia* 7780 infection in *G. mellonella* larvae. At the relatively low MOI of 10, AP3 was able to significantly (*P* < 0.0001) increase the survival rate of infected larvae even after 72 h post-infection. This is similar to the results obtained for lytic *Pseudomonas* phages (MOI = 100) examined by us previously (Olszak et al. 2015; Danis-Wlodarczyk et al. 2016). The application of temperate phages might result in possible undesirable consequences related to phage integration and potential increase in host virulence (Young and Gill 2015). However, a detailed analysis of phage AP3 genome against the database confirmed lack of toxin sequences, similarly to previous report about *Burkholderia* phages (Summer et al. 2007b). Furthermore, isolated phage-resistant clones were positive for AP3 genome acquisition as prophage or episome form of phage, and they showed no significant increase in bacterial pathogenicity compared to the parental strain in the *G. mellonella* infection model. AP3-integrated strains remained constant so virulence seems to be neither positively nor negatively affected. This agrees with previous reports (Summer et al. 2007a, b; Ronning et al. 2010) indicating there is no experimental evidence that *Burkholderia* virulence determinants arise from phage origin, except drug resistance to particular antibiotics. However, it is possible that the *Burkholderia* species collect prophages and prophage-like elements to acquire genes benefiting their survival in soil and rhizosphere environment (Summer et al. 2007a, b; Ronning et al. 2010). In general, the temperate phages condition horizontal dissemination of genes (HGT) by transduction and then phage conversion. It should be emphasized that lytic phages and bactericidal antibiotics are responsible for HGT as well by lysis product release as DNA fragments and indirectly by induction of temperate phages (Weinbauer 2004). Of course, the in vivo efficacy of these phages would need to be established. A major consideration is that the CF lung environment has a dynamic microbiome and it is not possible to predict the possible impact of each phage protein that could be an effector of BCC fitness in the lung. Nevertheless, our results show good lytic activity of the temperate AP3 phage with no evidence of increased virulence in lysogenic isolates in caterpillar model, suggesting that this phage is a potent lead against bacterial strains belonging to *B. cenocepacia* IIIA lineage, which are commonly isolated from CF patients.

## Electronic supplementary material

ESM 1 (PDF 1450 kb)
