# Supplementary material for: The temperate Burkholderia phage AP3 of the Peduovirinae shows efficient antimicrobial activity against B. cenocepacia of the IIIA lineage
Source: Appl Microbiol Biotechnol. 2016 Oct 21;101(3):1203–16. doi: 10.1007/s00253-016-7924-7 (PMC5247547; doi:10.1007/s00253-016-7924-7)
Supplement: Supplementary file 1 — (PDF 1450 kb) [file 253_2016_7924_MOESM1_ESM.pdf]

Applied Microbiology and Biotechnology

The temperate *Burkholderia* phage AP3 of the *Peduovirinae* shows efficient antimicrobial activity against *B. cenocepacia* of the IIIA lineage

Bartosz Roszniowski[1], Agnieszka Latka[1], Barbara Maciejewska[1], Dieter Vandenheuvel[2], Tomasz Olszak[1], Yves Briers[2,3], Giles S. Holt[4], Miguel A. Valvano[5], Rob Lavigne[2], Darren L. Smith[4] and Zuzanna Drulis-Kawa[1]*

[1]Institute of Genetics and Microbiology, University of Wroclaw, 51-148 Wroclaw, Poland

[2]Laboratory of Gene Technology, KU Leuven, Kasteelpark Arenberg 21, box 2462, 3001 Leuven, Belgium

[3]current affiliation: Department of Applied Biosciences, Ghent University, Valentin Vaerwyckweg 1, 9000 Gent, Belgium

[4]Applied Sciences, University of Northumbria, Ellison Building EBD222, Newcastle upon Tyne, NE1 8ST, United Kingdom

[5]Wellcome-Wolfson Institute for Experimental Medicine, Queen's University of Belfast; 97 Lisburn Rd. Belfast, UK BT9 7BL

*Corresponding author. Mailing address:
Zuzanna Drulis-Kawa
Institute of Genetics and Microbiology, University of Wroclaw,
Przybyszewskiego 63/77, 51-148 Wroclaw, Poland
Tel.: +48 71 375 62 90; Fax: +48 71 325 21 51
E-mail: zuzanna.drulis-kawa@uwr.edu.pl

Table S1. *Burkholderia* phage AP3 complete annotation

Ideogram location	Locus tag	Strand	Start position	End position	Residue length (nt)	Residue length (aa)	pI	Mw	Product	Homology	E-value (homology)	Motif	E-value (motif)
-	-	n/a	1	21	-	-	-	-	cosL	cosL of P2, phiCTX [*Enterobacteria* phage P2, *Pseudomonas* phage phiCTX]	-	-	-
-	-	+	214	240	-	-	-	-	Terminator	-	-	-	-
-	-	-	214	240	-	-	-	-	Terminator	-	-	-	-
1	vB_BceM_AP3_0001	-	265	591	21	108	5.04	11757.23	DNA-binding protein	putative helix-turn-helix transcriptional regulator [NP_536425]; [*Burkholderia* phage KS5]	7,00E-07	HTH_31	5.1e-10
2	vB_BceM_AP3_0002	-	575	829	326	84	9.56	10074.54	cytotoxic translational repressor of toxin-antitoxin stability system	hypothetical protein [NP_536426]; [*Burkholderia* phage phiE125]	5,00E-08	Plasmid_stabil	8.2e-08
-	-	-	837	865	-	-	-	-	promoter	-	-	-	-
3	vB_BceM_AP3_0003	-	878	1168	254	95	8.40	10803.14	hypothetical protein	-	-	-	-

4	vB_BceM_AP3_0004	+	1254	1967	290	237	5.53	27129.93	hypothetical protein	hypothetical protein [NP_536389]; [*Burkholderia* phage phiE125]	8,00E-97	DUF159	3.6e-27
5	vB_BceM_AP3_0005	-	1995	3047	713	350	8.39	39928.58	portal vertex protein	hypothetical protein [YP_004306410.1]; [*Burkholderia* phage KS5]	0.0	Phage_portal	1.2e-48
6	vB_BceM_AP3_0006	-	3047	4813	1052	588	7.62	67014.84	terminase ATPase subunit (P)	terminase endonuclease subunit (M) [YP_004306409.1]; [*Burkholderia* phage KS5]	0.0	Terminase_6	2.9e-99
-	-	+	4890	4918	-	-	-	-	promoter	-	-	-	-
7	vB_BceM_AP3_0007	+	4957	5775	1766	272	5.23	30377.79	phage capsid scaffolding protein	capsid scaffolding protein (O) [YP_004306408]; [*Burkholderia* phage KS5]	1.E-152	Phage_GPO	2.3e-94
8	vB_BceM_AP3_0008	+	5812	6831	818	339	5.68	38247.22	major capsid protein	capsid protein (N) [YP_004306407]; [*Burkholderia* phage KS5]	0.0	Phage_cap_P2	1.6e-135
9	vB_BceM_AP3_0009	+	6828	7514	1019	228	6.08	24781.15	small terminase subunit	terminase endonuclease subunit (M) [YP_004306407]; [*Burkholderia* phage KS5]	2,00E-147	Phage_term_smal	1.1e-49
10	vB_BceM_AP3_0010	+	7619	8101	686	160	5.45	18033.30	head completion protein	capsid completion protein (L) [YP_004306405]; [*Burkholderia* phage KS5]	4,00E-94	Phage_GPL	2.3e-59

11	vB_BceM_AP3_0011	+	8101	8355	482	84	11.30	10208.71	hypothetical protein	-	-	-	-
12	vB_BceM_AP3_0012	+	8352	8564	254	70	4.64	7764.96	tail protein	phage tail protein X [YP_004306403]; [*Burkholderia* phage KS5]	2,00E-40	Phage_tail_X	5.8e-26
13	vB_BceM_AP3_0013	+	8567	8941	212	124	5.65	12723.11	putative antiholin	putative antiholin [YP_004306402]; [*Burkholderia* phage KS5]	6,00E-78	Pfam-B_12172	9.5e-13
14	vB_BceM_AP3_0014	+	8941	9261	374	106	10.80	11313.51	putative holin	holin [YP_004306401]; [*Burkholderia* phage KS5]	-	DUF754	3.7e-25
15	vB_BceM_AP3_0015	+	9254	10054	320	266	8.82	28949.98	endolysin	endolysin [YP_004306400]; [*Burkholderia* phage KS5]	8,00E-160	DUF3380	1.9e-66
16	vB_BceM_AP3_0016	+	10051	10542	800	162	5.90	17108.27	i-spanin	Rz [YP_004306398]; [*Burkholderia* phage KS5]	4,00E-81	Pfam-B_8976	1.7e-05
17	vB_BceM_AP3_0017	+	10301	10495	491	64	10.82	6856.27	o-spanin	Rz1 [YP_004306399]; [*Burkholderia* phage KS5]	2,00E-27	-	-
18	vB_BceM_AP3_0018	+	10539	10949	194	136	5.01	15247.48	tail completion protein	tail completion protein (R) [YP_004306397]; [*Burkholderia* phage KS5]	5,00E-87	P2_Phage_GpR	1.1e-39
19	vB_BceM_AP3_0019	+	10949	11398	410	149	11.64	16784.37	virion morphogenesis protein	tail completion protein (S) [YP_004306396]; [*Burkholderia* phage KS5]	3,00E-83	Phage_tail_S	9.3e-39

20	vB_BceM_AP3_0020	+	11498	12061	449	187	12.17	22701.79	hypothetical protein	-	-	-	-
21	vB_BceM_AP3_0021	+	11556	12203	563	214	5.35	21960.18	baseplate assembly protein	phage baseplate assembly protein V [YP_008060505]; [*Burkholderia* phage ST79]	2,00E-60	Phage_base_V	8.5e-20
22	vB_BceM_AP3_0022	+	12200	12565	647	121	8.36	12984.93	baseplate assembly protein	baseplate assembly protein (W) [YP_004306393]; [*Burkholderia* phage KS5]	9,00E-69	GPW_gp25	4.7e-18
23	vB_BceM_AP3_0023	+	12562	13467	365	301	4.85	32936.68	baseplate assembly protein	baseplate assembly protein (J) [YP_004306392]; [*Burkholderia* phage KS5]	0.0	Baseplate_J	8.E-44
24	vB_BceM_AP3_0024	+	13460	14011	905	183	9.50	20388.48	tail protein	baseplate assembly protein (I) [YP_004306391]; [*Burkholderia* phage KS5]	1,00E-109	Tail_P2_I	8.9e-47
25	vB_BceM_AP3_0025	+	14017	17358	551	1113	5.66	117192.18	phage tail fiber protein	phage tail fiber protein [YP_008060509]; [*Burkholderia* phage ST79]	0.0	-	-
26	vB_BceM_AP3_0026	+	17371	17781	3341	136	5.78	15707.90	hypothetical protein	hypothetical protein [YP_008060510]; [*Burkholderia* phage ST79]	7,00E-34	-	-

27	vB_BceM_AP3_0027	+	17898	18236	410	112	5.31	12105.78	hypothetical protein	hypothetical protein [YP_008060511]; [*Burkholderia* phage ST79]	8,00E-11	-	-
28	vB_BceM_AP3_0028	+	18396	18578	338	60	9.92	6548.54	hypothetical protein	Com translational regulator [YP_004306388]; [*Burkholderia* phage KS5]	2,00E-30	Mu-like_Com	7.5e-15
29	vB_BceM_AP3_0029	+	18556	19305	182	249	7.70	27322.07	DNA methylase N-4	N-4/N-6 DNA methylase [YP_004306387]; [*Burkholderia* phage KS5]	5,00E-165	N6_N4_Mtase	2.5e-15
30	vB_BceM_AP3_0030	+	19417	20589	749	390	5.48	42611.04	tail sheath protein	tail sheath protein (FI) [YP_004306386]; [*Burkholderia* phage KS5]	0.0	Phage_sheath_1	6.8e-44
31	vB_BceM_AP3_0031	+	20619	21128	1172	169	4.85	18809.46	major tail tube protein	tail tube protein (FII) [YP_004306385]; [*Burkholderia* phage KS5]	0.0	Phage_tube	2.2e-70
32	vB_BceM_AP3_0032	+	21155	21472	509	105	4.91	11245.07	tail protein	tail protein (E) [YP_004306384]; [*Burkholderia* phage KS5]	1,00E-46	FluMu_gp41	4.2e-11
33	vB_BceM_AP3_0033	+	21472	21591	317	39	4.73	4693.32	P2 GpE family protein	tail protein (E+E') [YP_004306857]; [*Burkholderia* phage KS14]	1,00E-08	Phage_P2_GpE	3.2e-19
34	vB_BceM_AP3_0034	+	21588	24839	119	1083	10.38	114442.16	hypothetical protein	tail tape measure protein (T) [YP_004306429]; [*Burkholderia* phage KL3]	0.0	Pfam-B_7981	6.3e-07

35	vB_BceM_AP3_0035	+	24853	25281	3251	142	5.09	15761.10	P2 GpU family protein	tail protein (D) [YP_004306380]; [*Burkholderia* phage KS5]	0.0	Phage_P2_GpU	7.4e-47
36	vB_BceM_AP3_0036	+	25278	26432	428	383	9.07	41952.73	late control geneD protein	tail protein (D) [YP_004306380]; [*Burkholderia* phage KS5]	0.0	Phage_GPD	1.9e-60
37	vB_BceM_AP3_0037	+	26494	27246	1154	250	9.25	29164.58	glycosyl transferase	-	-	Glyco_transf_25	2.E-33
38	vB_BceM_AP3_0038	-	27368	28123	752	251	8.50	27039.51	alpha/beta hydrolase	hypothetical protein [YP_293723]; [*Burkholderia* phage phi52237]	2,00E-90	-	-
39	vB_BceM_AP3_0039	-	28178	28636	755	152	9.00	16088.92	XRE family transcriptional regulator	helix-turn-helix domain protein [YP_293722]; [*Burkholderia* phage phi52237]	7,00E-58	HTH_31	1.5e-11
-	-	+	28653	28681	-	-	-	-	promoter	-	-	-	-
40	vB_BceM_AP3_0040	+	28720	28956	458	78	11.14	8966.41	hypothetical protein	hypothetical protein [YP_004306376]; [*Burkholderia* phage KS5]	7,00E-40	-	-
41	vB_BceM_AP3_0041	+	28960	29238	236	92	7.71	9933.50	DNA binding protein	DNA binding protein [YP_004306375]; [*Burkholderia* phage KS5]	2,00E-61	-	-

42	vB_BceM_AP3_0042	+	29250	29498	278	82	9.23	9092.61	transcriptional regulator	transcriptional activator (Ogr) [YP_004306374]; [*Burkholderia* phage KS5]	1,00E-48	Ogr_Delta	6.6e-17
43	vB_BceM_AP3_0043	+	29586	29780	248	64	5.33	6933.96	hypothetical protein	hypothetical protein [YP_004306373]; [*Burkholderia* phage KS5]	4,00E-23	-	-
44	vB_BceM_AP3_0044	+	29785	29979	194	64	7.85	7327.39	hypothetical protein	hypothetical protein [YP_004306419]; [*Burkholderia* phage KS5]	4,00E-36	-	-
45	vB_BceM_AP3_0045	+	30023	30217	194	64	10.26	6892.42	hypothetical protein	hypothetical protein [YP_004306418]; [*Burkholderia* phage KL3]	2,00E-28	-	-
46	vB_BceM_AP3_0046	+	30222	30677	194	151	9.72	16971.35	hypothetical protein	-	-	-	-
47	vB_BceM_AP3_0047	+	30681	31043	455	119	6.90	12882.39	hypothetical protein	hypothetical protein [YP_004306417]; [*Burkholderia* phage KL3]	3,00E-64	-	-
48	vB_BceM_AP3_0048	+	31040	31288	362	82	6.22	8311.47	hypothetical protein	hypothetical protein [YP_004306416]; [*Burkholderia* phage KL3]	4,00E-47	-	-
49	vB_BceM_AP3_0049	+	31291	34083	248	930	6.97	102387.25	hypothetical protein	zinc finger CHC2-family protein [YP_004306415]; [*Burkholderia* phage KL3]	0.0	Pfam-B_9177	3.5e-35

50	vB_BceM_AP3_0050	+	34080	34691	2792	202	11.15	22043.72	baseplate J family protein	hypothetical protein [AIP84258]; [*Burkholderia* phage KS5]	9,00E-35		
-	-	+	34896	34925	-	-	-	-	promoter	-	-	-	-
51	vB_BceM_AP3_0051	+	34972	36048	611	358	10.30	40845.88	integrase	site-specific recombinase [YP_001165299]; [Ralstonia phage RSA1]	0.0	Phage_integrase	1.6e-36
-	-	n/a	36458	36478	1076	-	-	-	cosR	cosR of P2, phiCTX [*Enterobacteria* phage P2, *Pseudomonas* phage phiCTX]	-	-	-

Table S2. AP3 promoters and terminators discovered based on MEME analysis. Conserved motifs in promoters are noted in bold. Stem loops in terminators are underlined

**Location**	**Sequence**
Putative host promoters	
865 … 837	CCTTTACAT**TTGTGA**AAACTTATAATAAAACT**TATAAT**TATAAAATGC
4890 … 4918	CCAACATGCG**TTGCGA**CTCCGGTTCGAACTGCC**GAAACT**GGAATCCCTG
28653 … 28681	AAGTTTTCCG**TTGCAA**TTTGCCAAATTGGCAAT**TACACT**TAGCTTGTGC
34896 … 34925	TTTTCGACAG**TTGCTC**TGCAGCGGTACCTCGCCG**GAGAAT**GGATTCCGCG
Host Promoter	
E. coli consensus sequence	TTGACA-(N15-18)-TATAAT
Rho-independent terminators	
214…240	AAAAAAAAAAGCCGCCGGCACCGAAGTGCCTGGCGGCTTTTTGCTGC
240...214	GCAGCAAAAAGCCGCCAGGCACTTCGGTGCCGGCGGCTTTTTTTTTT

Table S3. Genome characteristics and pairwise identity of AP3 and selected BCC *P2likeviruses* based on NCBI BLASTN

**Phage**	**Genome length (bp)**	**CDS**	**G+C%**	**Nucleotide pairwise identity to AP3 (%)**	**Host**
AP3	36,499	50	64.04	-	*B. cenocepacia*
KS5	37,236	45	63.70	61.9	*B. cenocepacia*
KL3	40,555	52	63.22	49.5	*B. ambifaria*
φE202	35,741	48	65.42	46.7	*B. thailandensis*
BEK	37,631	57	68.82	48.4	*B. pseudomallei*
φX216	37,637	47	64.82	48.4	*B. pseudomallei*
φ52237	37,639	47	64.81	47.6	*B. pseudomallei*

Table S4. Detailed data of genes with highest homology to the AP3 tail fiber protein (TFP) versus bacteria

Host	TFP gene accession number	Location in host genome		Size (aa)	Homology to phage AP3 TFP	Notes
		Start	End			
B. cenocepacia D2AES	WP_027809284.1	21300	17956	1114	82%	WGS, contig 00300
B. cenocepacia PT15	WP_027812672.1	16583	19945	1120	77%	WGS, contig 00342
B. glumae 3252-8	WP_051071125.1	9639	6280	1119	76%	WGS
B. glumae BGR1	WP_043307224.1	1914720	1918094	1124	71%	Chromosome 1
B. gladioli UCD-UG CHAPALOTE	WP_036037003.1	288409	285392	1005	72%	WGS, scaffold 1.1
B. glumae NCPPB 3923	WP_039201646.1	6996	10013	1005	73%	WGS, contig 73
B. glumae LMG 2196	WP_045678863.1	1147723	1144616	1035	76%	Also known as *B. glumae* ATTC 33617, chromosome 1

Fig. S1. The comparison of 55-bp *cos* region of AP3 to the determined *cos* regions in *Enterobacteria* phage P2 (GenBank:NC_001895) and *Pseudomonas* phage phiCTX (GenBank:NC_003278.1): conserved sequence (marked yellow).

AP3 GCATGAGTGCATAAAAACCGTTCGTTTTTTGCGGGCAGGTGGGGCGGGGTCACAAC

P2 ATTAAGGTGCATTAAAACCGCCCCGTGAAGCGGGCGGGCGAGGCGGGGAAAGCAC

phiCTX CAGAAAGTGCAGAAAAAGAGGTCTATTTAGAGCGGAGGCGTGGCGGGGGGACGAC

Fig. S2. PFGE fingerprints of *B. cenocepacia* IIIA isolates sensitive to AP3 phage digested with *Xba*I enzyme. MM - MidRange PFGE Marker (New England Biolabs).

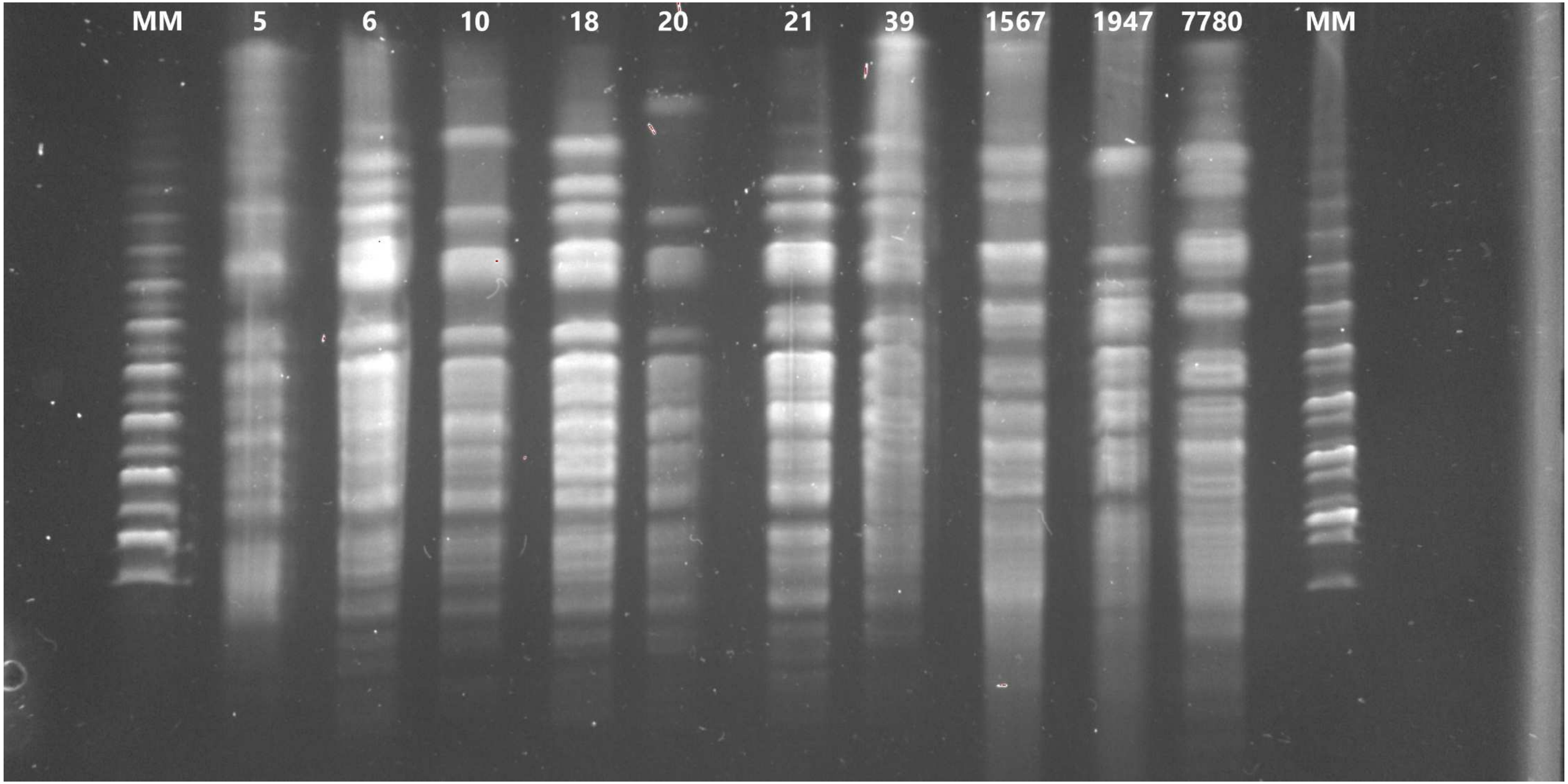

Fig. S3. Tail fiber amino acid sequence of *Burkholderia* phage AP3 versus known integrated and lytic *Burkholderia* phages (similarity ≥ 70%).

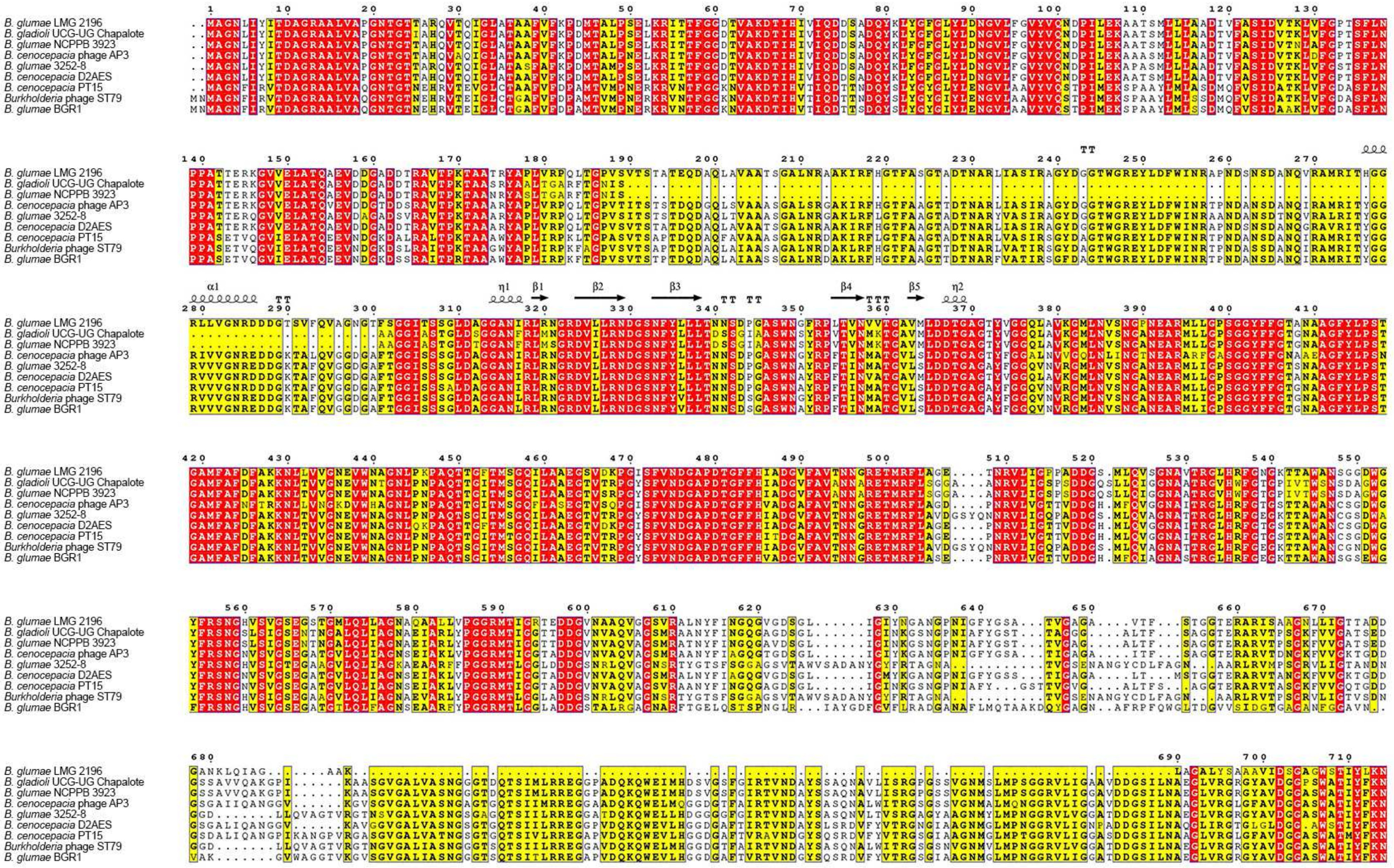

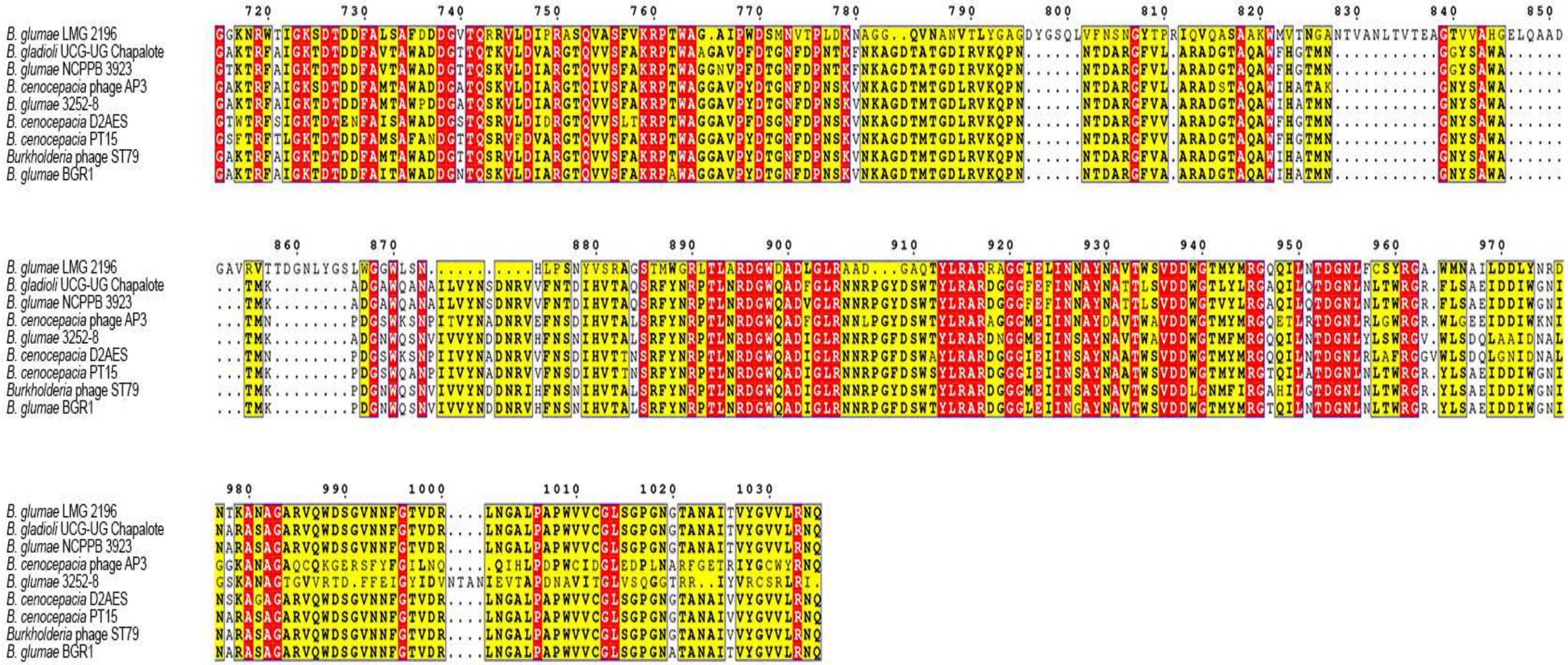
720 730 740 750 760 770 780 790 800 810 820 830 840 850
860 870 880 890 900 910 920 930 940 950 960 970
980 990 1000 1010 1020 1030
B. glumae LMG 2196
B. gladioli UCG-UG Chapalote
B. glumae NCPPB 3923
B. cenocepacia phage AP3
B. glumae 3252-8
B. cenocepacia D2AES
B. cenocepacia PT15
Burkholderia phage ST79
B. glumae BGR1

Fig. S4. Comparison of tail fiber amino acid sequence between *Burkholderia* phage AP3 and discussed *Burkholderia* phages.

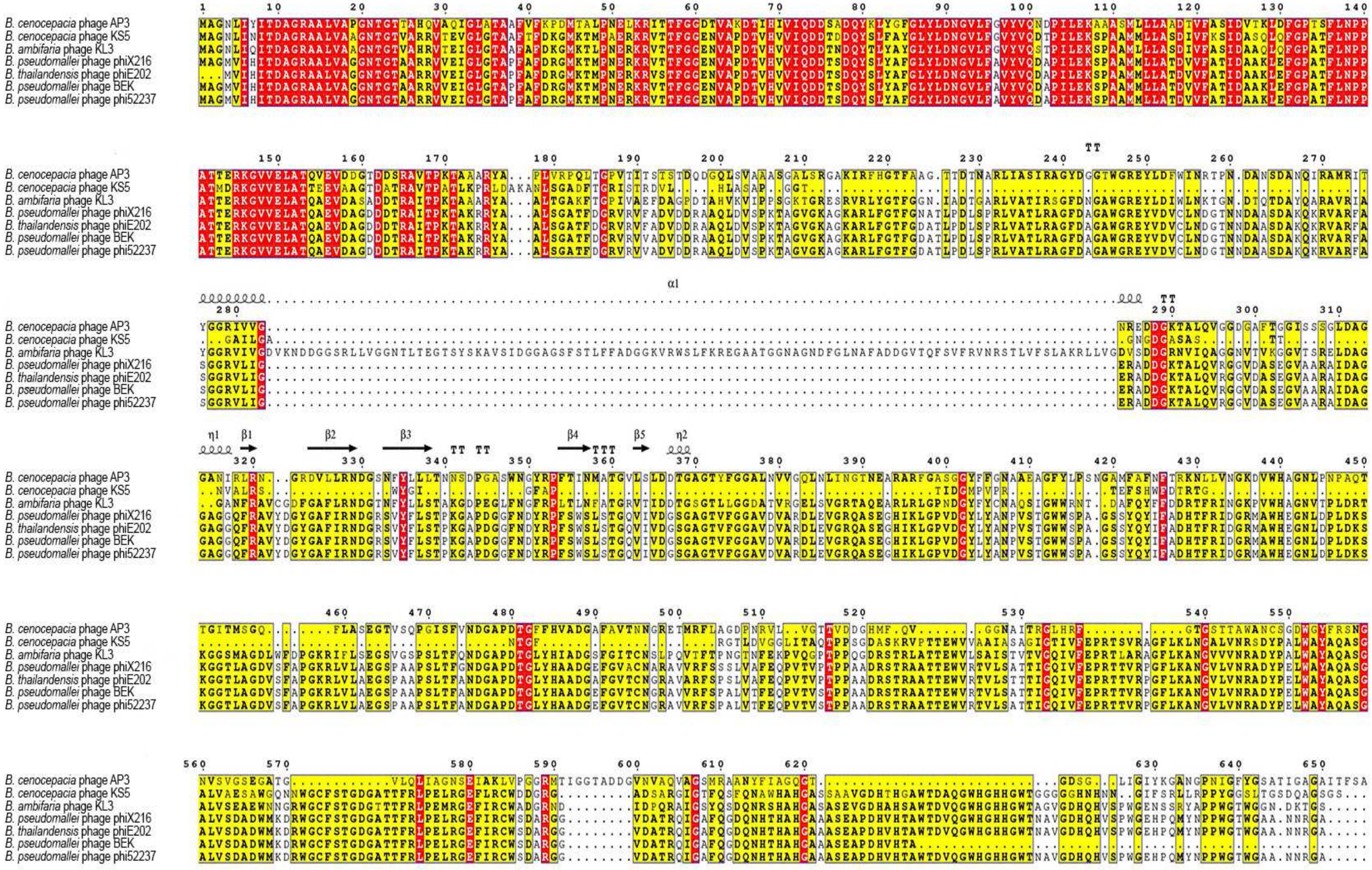

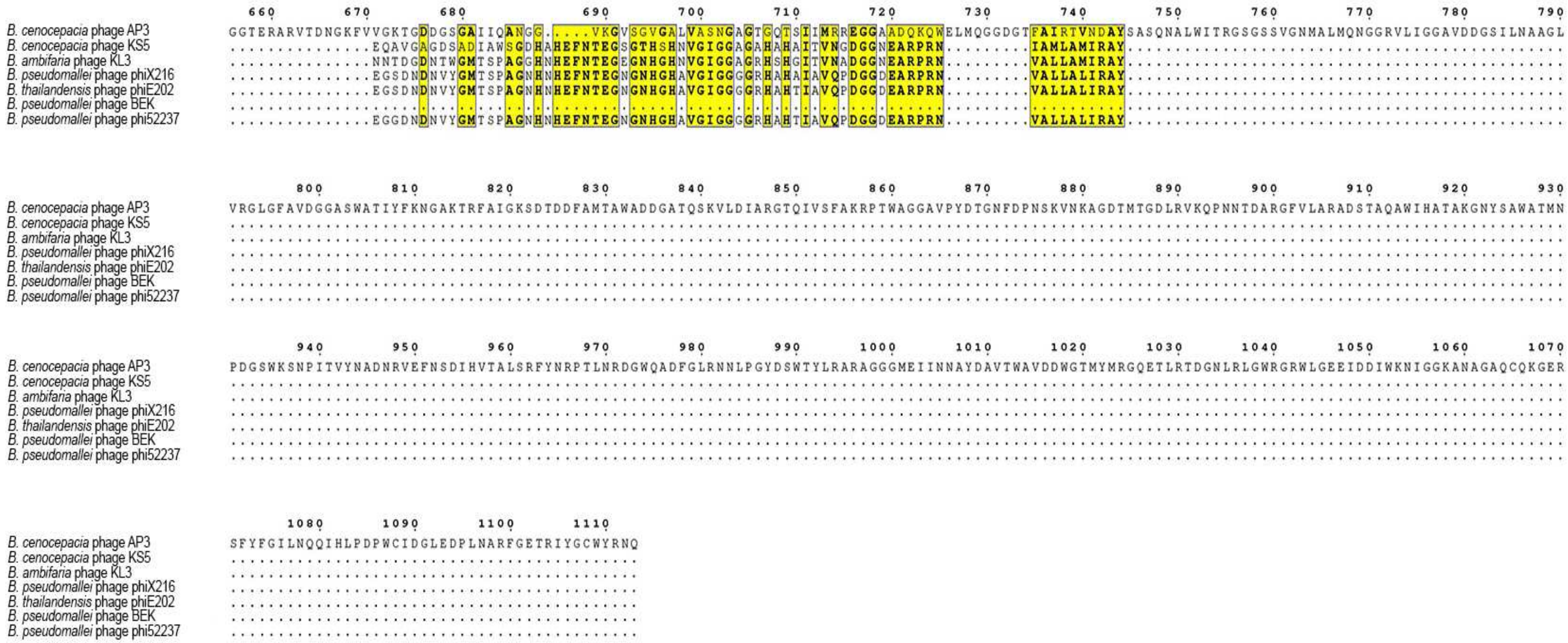

B. cenocepacia phage AP3
B. cenocepacia phage KS5
B. ambifaria phage KL3
B. pseudomallei phage phiX216
B. thailandensis phage phiE202
B. pseudomallei phage BEK
B. pseudomallei phage phi52237
